# Bacterial constipation: Mucin-degrading intestinal commensal bacteria cause constipation

**DOI:** 10.1080/19490976.2025.2596809

**Published:** 2026-02-18

**Authors:** Tomonari Hamaguchi, Noriaki Gibo, Misuzu Ohara, Mikako Ito, Tomoyuki Ogura, Jun-Ichi Takeda, Hiroshi Nishiwaki, Fei Zhao, Ryo Kinoshita-Daitoku, Masashi Hattori, Koji Nonogaki, Tetsuya Maeda, Kenichi Kashihara, Yoshio Tsuboi, Masaaki Hirayama, Mitsuhiro Fujishiro, Hiroki Kawashima, Kinji Ohno

**Affiliations:** a Division of Neurogenetics, Center for Neurological Diseases and Cancer, Nagoya University Graduate School of Medicine, Nagoya, Japan; b Department of Gastroenterology, Nagoya University Graduate School of Medicine, Nagoya, Japan; c Gibo Hepatology Clinic, Matsumoto, Japan; d Department of Pathophysiological Laboratory Sciences, Nagoya University Graduate School of Medicine, Nagoya, Japan; e Central Institute for Experimental Medicine and Life Science, Kawasaki, Japan; f Center for One Medicine Innovative Translational Research (COMIT), Institute for Advanced Study, Gifu University, Gifu, Japan; g Department of Bacteriology, Nagoya University Graduate School of Medicine, Nagoya, Japan; h Department of Gastroenterology, Yamashita Hospital, Ichinomiya, Japan; i Department of Gastroenterology, Daido Hospital, Nagoya, Japan; j Division of Neurology and Gerontology, Department of Internal Medicine, School of Medicine, Iwate Medical University, Iwate, Japan; k Okayama Neurology Clinic, Okayama, Japan; l Department of Neurology, Long-term Observation Research, Juntendo University, Tokyo, Japan; m Department of Occupational Therapy, Chubu University College of Life and Health Sciences, Kasugai, Japan; n Department of Gastroenterology, University of Tokyo, Tokyo, Japan; o Graduate School of Nutritional Sciences, Nagoya University of Arts and Sciences, Nisshin, Japan

**Keywords:** Bacterial constipation, Parkinson's disease, chronic idiopathic constipation, *Akkermansia muciniphila*, *Bacteroides thetaiotaomicron*, mucin degradation, sulfatase, gnotobiotic mice

## Abstract

The contribution of gut microbes to constipation remains mechanistically underexplored, despite constipation being one of the most prevalent gastrointestinal disorders. Here, we identify cooperative induction of constipation by two mucin-degrading gut commensals: *Akkermansia muciniphila* and *Bacteroides thetaiotaomicron*. In constipated patients with Parkinson’s disease (PD) and chronic idiopathic constipation (CIC), we observed that *A. muciniphila* and *B. thetaiotaomicron* were increased. Gnotobiotic mice colonized with either bacterium exhibited no constipation, whereas mice co-colonized with both bacteria developed constipation. Fecal mucins but not gastric mucins carry terminal sulfates. As fecal transcriptome of gnotobiotic mice suggested a sulfatase-dependent mechanism, we generated an anaerobic sulfatase-maturating enzyme (anSME)-deficient *B. thetaiotaomicron* strain that cannot catabolize the terminal sulfates of mucins. In the absence of anSME, constipation was ameliorated in co-colonized gnotobiotic mice. The synergic effect of the two bacteria is in accordance with our observation that *A. muciniphila* alone and constipation are not correlated in humans. As a bunch of intestinal bacteria other than *B. thetaiotaomicron* also catabolize mucin sulfates, they may substitute for *B. thetaiotaomicron* in patients with constipation. We propose bacterial constipation, in which cooperative degradation of colonic mucins by sulfatases and glycosylases by two commensal bacteria reduces lubrication and induces fecal dehydration, leading to the development of constipation. Targeting microbial sulfatase activity may be a promising therapeutic approach for patients with bacterial constipation.

## Introduction

Constipation, defined as fewer than three bowel movements per week, is a common gastrointestinal disorder. It typically results from prolonged colonic transit, impaired rectal evacuation, or pelvic floor dysfunction, leading to hard, dry stools due to excessive water absorption.^1^ Constipation is frequently observed in neurodegenerative conditions, such as Parkinson’s disease (PD), and in functional disorders such as chronic idiopathic constipation (CIC).^1-3^ Constipation substantially impairs quality of life, imposes a considerable burden on health-care systems, and requires long-term management.

In PD, constipation often precedes motor symptoms by 10 to 20 years. It is thought to result from neurodegeneration within the enteric nervous system, thereby impairing gut motility.^4-7^ However, dopaminergic therapies and peripherally acting DOPA decarboxylase inhibitors provide little benefit for bowel dysfunction, indicating that PD-related constipation may involve mechanisms beyond dopaminergic neurodegeneration.^8^^,^^9^ In CIC, defined as constipation without secondary causes, conventional laxatives or prokinetic agents fail to provide durable symptom relief.^10^ These therapeutic limitations highlight the need to explore alternative mechanisms underlying constipation, beyond neural or motility dysfunction, with growing attention to the role of gut microbiota.

Notably, increased *A. muciniphila* abundance has been consistently reported in patients with PD across various countries.^11^^,^^12^ *A. muciniphila* is also associated with firmer stool and slower colonic transit, and its abundance has been linked to gut dysbiosis in constipation cohorts.^13-17^ Likewise, *Bacteroides* species have been reported to be abundant in the colonic mucosa of constipated patients,^13^^,^^18^ although their fecal abundance varies across studies.^14^^,^^19^^,^^20^ Together, these observations raise the possibility that gut microbial composition and function contribute to constipation in both PD and CIC.

Mucin is a glycoprotein that forms the colonic mucus layer. It plays a crucial role in protecting the intestinal lining and maintaining hydration by forming a gel-like barrier that shields the epithelium from mechanical stress and dehydration.^21-23^ Unlike the stomach and small intestine, colonic mucin carries terminal sulfates.^24^^,^^25^ While gastric mucin reduction has been reported in CIC patients,^26^^,^^27^ colonic mucin levels in constipation remain unexplored. In addition, the effects of colonic mucin metabolized by intestinal bacteria on constipation have not been investigated in either humans or animal models. These observations raise the possibility that specific microbial functions, rather than motility defects alone, may underlie constipation in a subset of patients.

Gut microbiota can modify mucus structure by breaking down mucin glycans,^28^^,^^29^ which may alter the mucus barrier integrity and composition.^22^^,^^30^ Among them, *A. muciniphila* and *Bacteroides* spp. are two major bacterial groups that are involved in mucin metabolism. However, *A. muciniphila* is unable to degrade sulfated colonic mucins, as it lacks sulfatases that are necessary for detaching the terminal sulfates from mucin oligosaccharides.^31^ In contrast, *Bacteroides* spp., particularly *Bacteroides thetaiotaomicron*, which is one of major species in *Bacteroides* spp. in human feces, possess versatile glycan-degrading capacities, including colonic mucin.^32-36^ Notably, some *Bacteroides* spp. express sulfatases, which allows the removal of terminal sulfate residues, thereby facilitating subsequent degradation of mucin glycans.^31^ Since desulfation can be a rate-limiting step in mucin degradation, we hypothesize that *Bacteroides*-mediated mucin desulfation enhances glycosidase activity in other gut microbes, including *A. muciniphila*, by enabling their access to mucins.^37-41^ Such cooperative mucinolysis may accelerate mucus breakdown and contribute to the development of constipation.

Here, we investigated this hypothesis by combining human microbiota profiling with gnotobiotic mouse models. Specifically, we analyzed gut microbiota in patients with PD and CIC, as well as in healthy controls. We established mono- and co-colonized gnotobiotic mice with *A. muciniphila* and/or *B. thetaiotaomicron*. Within the co-colonization setting, we further compared mice colonized with wild-type *B. thetaiotaomicron* and those colonized with *ΔanSME* mutant strains, which lack the anaerobic sulfating maturation enzyme, in order to specifically evaluate the contribution of this enzyme to host-microbe interactions. Through this approach, we aimed to clarify how cooperative interactions between mucin-degrading bacteria may influence stool mucin levels, hydration, and ultimately contribute to the pathophysiology of constipation.

## Materials and Methods

### Approvals of human and animal studies

Human studies were approved by the Ethical Review Committees (ERC) of the Nagoya University (approval #2016−0151), Iwate Medical University (approval #H28−123), Okayama Kyokuto Hospital (approval #kyoIR−2016002), Fukuoka Medical College (approval #2016M027), Daido Hospital (approval #ECD2019−011), and Yamashita Hospital (approval #YEC 19−03). Written informed consent was obtained from all participants. Animal studies were approved by the Animal Care and Use Committee of the Nagoya University (approval #M240271−001). All the human and animal studies were performed in accordance with relevant guidelines.

### Human subjects

Participants of PD patients and controls were identical to those in our previous studies.^11^^,^^42^^,^^43^ CIC patients were newly recruited for this study. Participants of CIC patients were age-matched to those of PD patients and controls. To ensure lack of any organic disease, blood tests and a colonoscopy were performed in all the CIC patients, but not in PD patients.

### Fecal samples and DNA isolation

A fecal sample was collected at the participant’s home and transported to Nagoya University while kept at 0 °C with ice packs in a thermal insulation jar as previously described.^11^ The fecal samples were freeze-dried using a freeze-dryer (FDU−2110, EYELA, Shanghai, China).^44^ Wet and dry fecal weights were recorded, and fecal moisture content (% wet weight) was calculated using the following formula:

$$\text{Moisture content}\;(\%)=\frac{\text{wet weight}-\text{dry weight}}{\text{wet weight}}\times 100$$

DNA was extracted from 20 mg of freeze-dried (FD) feces using the QIAamp PowerFecal DNA Kit (QIAGEN, Hilden, Germany) following the instructions of the manufacturer.^44^ The protocol was partially modified to use Lysing Matrix E Beads (MP Biomedicals, Irvine, CA, USA) with FastPrep−24 5G (MP Biomedicals) for three cycles at 6.0 m/s for 60 s instead of vortex mixing.

### 16S rRNA sequencing

Analysis of 16S rRNA sequencing was performed as previously described.^11^ Briefly, the V3-V4 hypervariable region of the bacterial 16S rRNA gene was amplified by primer 341F, 5’-CCTACGGGNGGCWGCAG−3’ and primer 805 R, 5’-GACTACHVGGGTATCTAATCC−3’. Paired-end sequencing of 300-nucleotide fragments was performed using the MiSeq reagent kit V3 on a MiSeq system (Illumina). Taxonomic analysis was performed with QIIME2.^45^ Operational taxonomic units (OTUs) were generated using DADA2,^46^ and the SILVA taxonomy database release 132^47^ was used for taxonomic identification. Eighty genera with relative abundances higher than 0.001 in controls, PD, and CIC were subjected to the following analysis. As the numbers of reads per sample were 66,064 ± 19,031 (mean and SD), the cutoff value of 0.001 eliminated genera with 66 reads or less on average.

### Determination of bacterial counts by hydrolysis probe quantitative real-time PCR (qPCR)

qPCR was performed using the Takara Thunderbird Probe qPCR Mix (Takara Bio) in conjunction with a hydrolysis probe specific to each bacterium. PCR primers and hydrolysis probes are indicated in Supplementary Table S1. PCR amplifications were performed using a LightCycler 480 Real-Time PCR System (Roche) with the following cycling conditions: Initial denaturation at 95 °C for 30 s, followed by 40 cycles of denaturation at 95 °C for 5 s and annealing/extension at 60 °C for 30 s. The abundance of total bacteria was examined by universal 16S rRNA primers, and the relative abundance of each bacterium was calculated.

### Loperamide-induced constipation study

C57BL/6N male mice at seven weeks of age in SPF were randomly assigned to either the control group (*n* = 10) or loperamide-induced constipation group (*n* = 11). Mice had *ad libitum* access to drinking water and CE−2 standard chow (CLEA). For the loperamide group, water contained 0.06 mg/mL loperamide, a *μ*-opioid receptor agonist targeting the myenteric plexus of the large intestine, and was provided continuously for six weeks. Based on average daily water intake (~5 ml/mouse), the estimated dose corresponded to ~0.3 mg/day (~10−15 mg/kg/day).^48^ Control mice took tap water.

Fecal samples were collected at baseline and at week six. DNA was extracted and analyzed by qPCR as described above. Constipation phenotypes were evaluated at 8 and 14 weeks of age using metabolic cages (Tecniplast) to measure fecal pellet count, fecal moisture content, and food/water intake. Outcome assessors were blinded to group assignment. Fecal samples collected at these time points were further used for bacterial abundance quantification by qPCR, as indicated in the figures.

### Bacterial culture and growth conditions

*A. muciniphila* (JCM 30893) and *B. thetaiotaomicron* (JCM 5827^T^) were obtained from the Riken BioResource Research Center. They were cultured in Brain Heart Infusion (BHI) medium (Shimadzu) at 37 °C in an anaerobic workstation chamber (Concept 400-M, Baker Ruskinn) that was filled with a gas mixture of 5% H_2_, 5% CO_2_, and 90% N_2_. The medium for *A. muciniphila* was supplemented with 0.1% porcine gastric mucin (Fujifilm Wako). *E.coli* strain S17-λ*pir*^49^ that was capable of conjugating with other bacteria was obtained from the National BioResource Project at the National Institute of Genetics. *E. coli* strain S17-λ*pir* was cultivated aerobically in LB medium at 37 °C.

### *In vitro* growth assay with loperamide

*A. muciniphila* and *B. thetaiotaomicron* were cultured in the presence or absence of loperamide. Bacteria were grown anaerobically in BHI supplemented with 0.5% porcine gastric mucin (Fujifilm Wako) at 37 °C. Loperamide (Fujifilm Wako) was dissolved in 100% DMSO and added to the culture medium at concentrations of 0.1, 1, and 10 µM with 0.1% DMSO. Optical density at 600 nm (OD_600_) was measured every 2−3 hours. Control bacteria were cultured under 0.1% DMSO. Each condition was tested in six biological replicates.

### Fiber-deprivation-induced constipation study

C57BL/6N SPF male mice at six weeks of age were randomly assigned to either the fiber-supplemented group (*n* = 6) or the fiber-deprived group (*n* = 6). Mice in the fiber-supplemented group had *ad libitum* access to AIN93M diet (CLEA) for 1 week. In contrast, mice in the fiber-deprived group had *ad libitum* access to fiber-free AIN93M diet lacking 5% alpha cellulose for 1 week, which was prepared by CLEA. After 1 week, constipation phenotypes were blindly evaluated using metabolic cages (Tecniplast) by measuring fecal pellet count/weight, food/water intake, and urinary output. Fecal DNA was extracted and the bacterial abundances were analyzed by qPCR as described above.

### Generation of germ-free and gnotobiotic mice

Altered Schaedler Flora (ASF, Taconic Bioscience) is comprised of eight defined bacteria, and is commonly used as a control in gnotobiotic mouse models.^50^ Germ-free IQI/Jic mice were colonized with ASF at the Central Institute for Experimental Medicine and Life Science (CIEM), and were transferred to Nagoya University. C57BL/6NJcl mice in SPF conditions were purchased from CLEA, and were made germ-free. Fecal samples of IQI/Jic mice were transplanted into germ-free C57BL/6N mice to make ASF C57BL/6N mice.

Germ-free ICR mice were purchased from Sankyo Labo Service Corp. To generate germ-free C57BL/6N pups, germ-free ICR dams served as foster mothers.^51^ Two days after mating a pair of germ-free ICR mice, a pair of SPF C57BL/6N mice were mated. The ICR mother delivered and reared her pups for 2 days, then the ICR pups were switched to the C57BL/6N pups that were sterilized as follows. To control the delivery date, the C57BL/6N mother was subcutaneously injected with 100 µl LUTEUM DEPOT (ASKA Pharmaceutical) for two consecutive days before the expected delivery date. On the expected delivery date, the uterus of the C57BL/6N mother was aseptically isolated after cervical translocation. It was transferred into a sterile flexible film isolator (ICM) after shortly soaking the uterus in water containing 0.33% MIKRO QUAT (Ecolab) and 8% ethanol to ensure sterility. The pups were taken out of the uterus, and any membranes or fluids from the pup's mouth and nose were immediately removed using a sterile cloth. The pup's chest and back were gently rubbed to stimulate breathing.

All mice were allowed to access food and water *ad libitum*. Sterile versions of bedding, drinking water, and CL−2 animal pellets irradiated at 50 kGy were purchased from CLEA. Environmental conditions were controlled at 23 ± 2 °C with a 12-hour light/dark cycle. Gnotobiotic mice were created by a single oral gavage of 100 µl liquid culture containing each anaerobe at OD_600_ = ~0.7, corresponding to ~2 × 10^8^ CFU/ml at age 5−7 weeks. To assess bacterial colonization levels in gnotobiotic mice, fecal DNA was extracted and quantitative PCR (qPCR) was performed using species-specific primers for *A. muciniphila* and *B. thetaiotaomicron* (Suppl. Table S1) in one week after gavage.

Surface samples inside the isolator and fecal pellet samples were collected. The samples were cultured in Thioglycollate (TGC) broth and on Potato Dextrose Agar (PDA) to ensure a bacteria-free and fungi-free environment within the isolator.

### Fecal mucin measurement

Fecal mucin contents per wet weight in humans and mice were blindly quantified by Fecal Mucin Assay Kit (Cosmo Bio) according to the manufacturer’s instructions. The mucin assay quantified mucin-derived O-glycans that were released under alkaline conditions by *β*-elimination and were fluorescently labeled at their reduced ends.^52^ This method allowed detection of mucin glycans while minimizing the detection of microbial or dietary glycans.^53^^,^^54^

### Fluorescent staining of mouse fecal samples

Fresh fecal pellets were fixed using Carnoy’s solution and embedded in paraffin.^55^ The slices of pellets were stained with iFluor 488-Wheat Germ Agglutinin (WGA) (AAT Bioquest), Ulex Europaeus Agglutinin I (UEA I) DyLight 649 (Vector Laboratories), and anti-Muc2 antibody [EPR23479−47] (Abcam) along with Goat anti-Rabbit IgG (H + L) Cross-Adsorbed Secondary Antibody Alexa Fluor 594 (Thermo Fisher Scientific). The images were acquired using a BZ-X800 microscope (Keyence). Signal intensities were quantified by Fiji version 2.1.4.0 by personnel blinded to group assignment. Human fecal samples were not subjected to fluorescent staining due to loss of microscopic integrity during transport from home to the laboratory.

### RNA-seq analysis for mouse colonic tissues

Distal colon tissues were dissected and rinsed in ice-cold PBS, and total RNA was extracted using the RNeasy Mini Kit (Qiagen) with on-column DNase treatment (*n* = 5 per group). RNA quality was confirmed by Bioanalyzer (Agilent, RIN ≥ 7). Strand-specific poly(A)-selected libraries were prepared using the VAHTS Universal V8 RNA-seq Library Prep Kit (Illumina-compatible; Azenta Life Sciences) and sequenced on a NovaSeq 6000 (Illumina, 2 × 150 bp, ~6 Gb/sample). Adapter trimming and quality control were performed by Azenta. Clean reads were aligned to the mouse reference genome (GRCm39) with HISAT2^56^ (Galaxy v2.2.1),^57^ gene-level counts were obtained by featureCounts^58^ (Galaxy v2.1.1), and differential expression analysis was conducted with DESeq2^59^ (Galaxy v2.11.40.8). Data visualization was performed using iDEP2.1.^60^

### Fluorescent staining of mouse colonic tissues

Distal colonic segments were fixed in 4% paraformaldehyde (PFA) and embedded in paraffin. Sections were stained with iFluor 488–WGA (AAT Bioquest). Images were acquired using an APX100 microscope (Evident Scientific). The area and number of goblet cells were blindly quantified using Fiji,^61^ while the goblet cell regions were automatically segmented by the Labkit plugin (https://imagej.net/plugins/labkit/).^62^

### Metabolic cage analysis

The metabolic activities of a mouse were evaluated using a metabolic cage (Tecniplast) that allowed the collection of feces and urine and the measurement of food and water intake in 24 h (*n* = 5−6 mice per group). The plastic parts of metabolic cage were disinfected by a spray of 2% peracetic acid (Mitsubishi Gas Chemical Trading) in the connecting sleeve attached to a flexible film isolator overnight, while the metallic parts were autoclaved in a sterile drum at 132 °C for 30 min. Mice were individually housed in the metabolic cages for acclimatization for 24 h before data acquisition to minimize stress-induced alterations in behaviors and metabolisms. Prior to the assay, pre-weighed food pellets and pre-weighed water were provided to each cage. Fecal pellets were counted in the collection column for 24 h. The collected feces were freeze-dried and fecal moisture contents (% wet weight) were calculated as stated for human fecal samples. The urine was measured using a disinfected graduated cylinder. In 24 h, the remaining food and water were weighed and measured, respectively.

### FITC-dextran permeabilization assay

ICR mice were fasted for 4 h before and during the experiment. Mice had transanal administration of 5 ml/kg of FITC-dextran (20 mg/ml solution). After 3 h, blood sample was obtained, and the plasma was diluted to 1:2 with PBS. FITC-dextran permeability was assessed by measuring 100 µl of plasma samples alongside 100 µl of serially diluted standards in a black 96-well plate by personnel blinded to group assignment.

### RNA-seq analysis for bacteria

Total RNA was extracted from fresh fecal samples (*n* = 4 mice per group) using the RNeasy PowerMicrobiome Kit (Qiagen) following the manufacturer’s protocols. The quality and quantity of the extracted RNA were assessed using the NanoDrop spectrophotometer (Thermo Fisher Scientific) and High Sensitivity RNA ScreenTape (Agilent Technologies). RNA-seq libraries were prepared using the Ribo-Zero Plus rRNA Depletion kit (Illumina) following the manufacturer's instructions. The libraries were sequenced on the NovaSeq 6000 (Illumina) at Macrogen to generate 150 bp paired-end reads. Low-quality reads and adapter sequences were trimmed using fastp v0.23.4 with −detect_adapter_for_pe -*n* 10 -l 20.^63^ Trimmed reads were mapped to the complete genomic sequence (NCBI RefSeq Assembly ID, GCF_009731575.1) of *A. muciniphila* (JCM 30893) using Bowtie2 v2.5.3 with default parameters for end-to-end alignment.^64^ The read count of each gene was obtained from the alignment using featureCounts v2.0.6 with -pC -t CDS -g gene_id.^58^ Read counts were processed and normalized using DESeq2.1.^59^ Differential expression analysis between *A.m.* and *A.m & B.t.* groups was conducted using DESeq2.1, and additional visualization was performed using iDEP2.0.^60^ Genes with an adjusted *p*-value < 0.05 and |log_2_ fold change| > 1.0 were considered to be significantly different.

### Generation of an *anSME* deletion mutant of *Bacteroides thetaiotaomicron*

anSME is a key enzyme required to mature sulfatases in *B. thetaiotaomicron*.^65^^,^^66^ We created an allelic deletion mutant of *anSME* essentially according to the previously reported method.^67^ Complete genomic sequence of *B. thetaiotaomicron* (DSM 2079) was obtained with the NCBI assembly ID of GCF_014131755.1. Two ~1-kb fragments upstream and downstream of the *anSME* gene were amplified by PCR and were cloned into the pGEM-T Easy vector (Promega), respectively. The inserts were again amplified by PCR, while 15 nucleotides at the 5’ end of the downstream fragment were artificially added at the 3’ end of the upstream fragment to serve as a homologous region for recombination. The two PCR products were subcloned into the pLGB−30 vector via In-Fusion system (Takara) to generate pLGB30-*ΔanSME.* pLGB30-*ΔanSME* was transformed into the donor *E.coli* strain S17-λ*pir*. *E. coli* was cultured in LB medium until OD_600_ became 0.2 to 0.6. Similarly, *B. thetaiotaomicron* was cultured in BHIS medium that was constituted of BHI medium, 5 mg/L hemin, and 2.5 g/L vitamin K1, until OD_600_ became 0.1 to 0.2. Then, the two bacteria were mixed and pelleted by 9,000 × *g* for 10 min. To mate the two bacteria, the pellet was resuspended in 100 µl BHIS liquid medium, directly spotted onto a Columbia blood agar plate, and incubated at 37 °C aerobically for 15 to 18 h. The bacteria from the spot were streaked on a Columbia blood agar containing 30 µg/ml kanamycin to eliminate *E. coli*, while *B. thetaiotaomicron* is resistant to kanamycin. Colony PCR was performed to verify the knockout of *anSME*. Positive colonies were streaked on a Columbia blood agar plate containing 10 mM rhamnose to activate *ssBfe1* in pLGB30 to eliminated pLGB30-carrying *B. thetaiotaomicron* and 30 µg/ml kanamycin to eliminate *E. coli*. Only *anSME*-deficient *B. thetaiotaomicron* that lost pLGB30 was expected to grow on the plate. After 3 to 4 days, colony PCR was performed to verify the knockout of *anSME* again. PCR primers are shown in Supplementary Table S2.

### *In vitro* growth assay with sulfated glycans

The composition of the minimal medium and the growth assay protocol were adapted from Benjdia et al.^66^ Briefly, minimal medium was supplemented with 0.5% (w/v) glucose, 0.5% (w/v) chondroitin sulfate (Sigma-Aldrich), or 0.5% (w/v) heparin (Sigma-Aldrich) as the sole carbon source. Bacterial growth was monitored by measuring optical density at 600 nm (OD₆₀₀) every 15 min.

### Statistical analysis

For parametric data, multiple groups were analyzed by one-way ANOVA followed by Tukey’s post hoc test or two-way ANOVA followed by Sidak’s multiple comparisons test. For nonparametric data like bacterial abundances, pairs were analyzed by Mann-Whitney test, and multiple groups were analyzed by Kruskal-Wallis test followed by Dunn’s post hoc test or by Steel test. Spearman’s rank correlation coefficients (*ρ*) were calculated to assess relationships between fecal mucin content (mg/g), fecal moisture content (%), stool frequency (/week), and the relative abundance of *A. muciniphila* (log_10_-transformed) within each subject group (PD, CIC, and controls). The effect of loperamide on bacterial abundances was analyzed using a two-way repeated-measures ANOVA with Sidak’s multiple comparisons test. The analysis was all performed on Prism 10 (GraphPad Software). For the choice of analytical methods, we followed the recommendations of Prism 10 and confirmed the appropriateness of the choice on literature. Statistical significance was defined as *p* < 0.05.

## Results

### *A. muciniphila* and *B. thetaiotaomicron* are increased and fecal mucins are decreased in patients with Parkinson’s disease (PD) and chronic idiopathic constipation (CIC)

**Figure 1.**
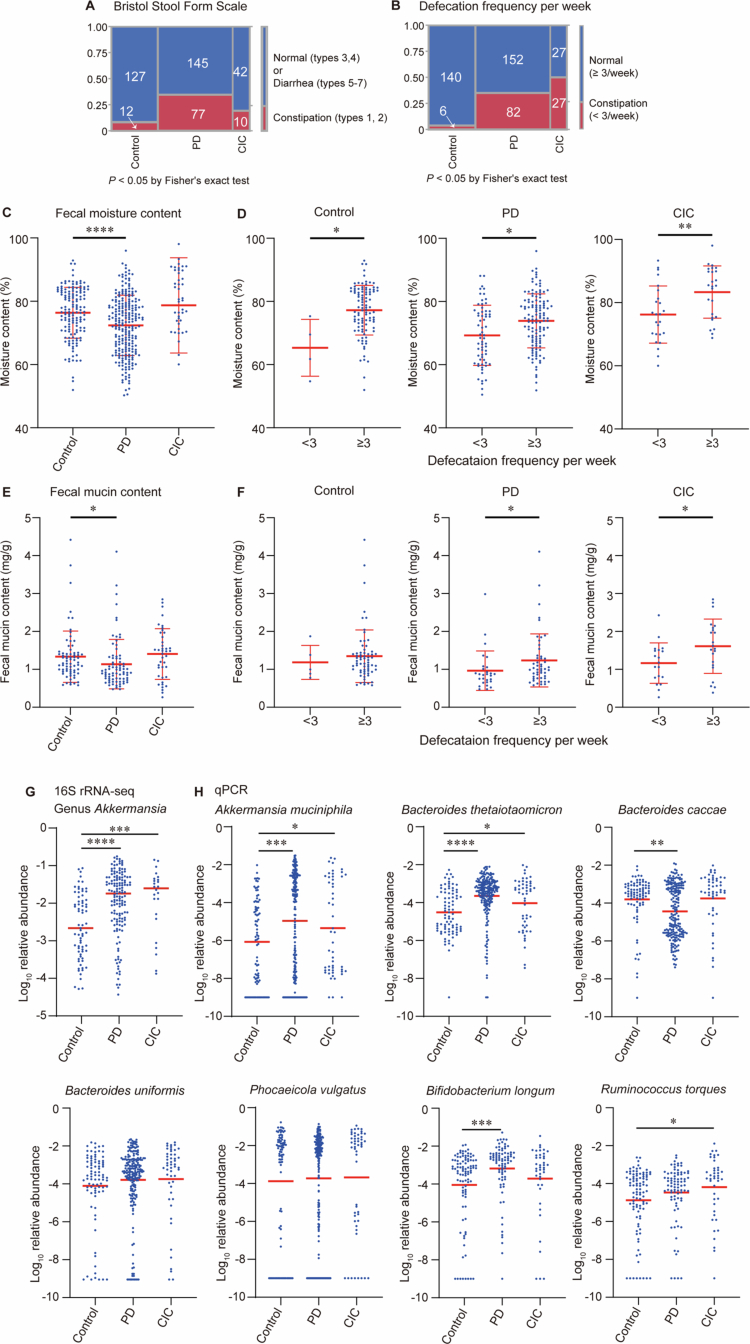
Fecal and defecation features and qPCR of seven glycan-cleaving intestinal bacteria in healthy controls and in patients with Parkinson’s disease (PD) and chronic idiopathic constipation (CIC). Bristol Stool Form Scale (A) and defecation frequency per week (B) in controls, PD, and CIC. The numbers of individuals and *p*-values by Fisher’s exact test are indicated in cells and at the bottom, respectively. Fecal moisture contents in controls, PD, and CIC (C), and in constipated (<3 defecation/week) and non-constipated (≥3 defecation per week) subjects in each disease category (D). Fecal mucin contents in controls, PD, and CIC (E), and in constipated and non-constipated subjects in each disease category (F). (G) Relative abundance of genus *Akkermansia* in controls, PD, and CIC in 16S rRNA sequencing analysis. (H) Relative abundance of seven experimentally proved glycan-cleaving intestinal bacteria by hydrolysis probe-based qPCR. (C–F) Mean and SD are indicated. (G, H) Medians are indicated. *p < 0.05, **p < 0.01, ***p < 0.005, and ****p < 0.001 by Mann-Whitney test (D, F) and Kruskal-Wallis test followed by Dunn’s post hoc test (C, E, G, H).

We collected fecal samples from 231 PD patients, 54 CIC patients, and 147 healthy controls. Functional constipation was postulated in PD patients and was confirmed in CIC patients. The demographic and clinical features of the participants are summarized in Table 1. The sex, age, and BMI were not significantly different in the groups. The Bristol Stool Form Scale, representing the fecal forms, was lower in PD and CIC compared to controls, indicating that feces were harder in PD and CIC (Figure 1A). As expected, the defecation frequency was lower in PD and CIC compared to controls (Figure 1B). We then estimated fecal moisture contents in controls, PD, and CIC by weighing fecal samples before and after freeze drying.^44^ Fecal moisture contents were lower in PD, but not in CIC, compared to controls (Figure 1C). However, when subjects were divided into the constipated (<3 bowel movements per week) and non-constipated (≥3 bowel movements per week) subgroups, the constipated subgroups had significantly lower fecal moisture content in all the groups (Figure 1D). Most constipated patients were on laxatives, and laxatives were likely to have increased fecal moisture content. This might account for lack of the difference in moisture contents between controls and CIC (Figure 1C), as well as in moisture contents in the subgroup analysis (Figure 1D).

**Table 1.** Demographic and clinical features of participants.

	Controls	PD	CIC	*P* < 0.05
# Subjects (M/F)	146 (71/75)	231(96/135)	54 (22/32)	n.s.^a^
Age (years)^b^	68.5 ± 9.7	68.5 ± 8.5	67.1 ± 12.8	n.s.^c^
BMI^b^	22.8 ± 3.1	21.6 ± 3.1	22.4 ± 3.1	n.s.^c^
Bristol Stool Form Scale^b^	3.9 ± 0.9	3.3 ± 1.4	3.8 ± 1.5	HC-PD^c^
Stool frequency (/week)^b^	8.1 ± 4.4	4.6 ± 3.9	4.2 ± 4.0	HC-PD, HC-CIC^c^

PD, Parkinson’s disease; CIC, chronic idiopathic constipation; M, males; F, females; and n.s., no significance.

a Fisher’s exact test.

b Mean and SD are indicated.

c One-way ANOVA followed by Tukey’s post hoc test. Statistically different pairs are indicated by a hyphen.

Because mucin can retain a large amount of water,^68^ we next examined the fecal mucin contents by quantifying mucin-derived O-glycans. Fecal mucin contents were lower in PD, but not in CIC, compared to controls (Figure 1E). Spearman’s correlation coefficient between fecal mucin contents and fecal moisture contents was 0.15 (Suppl. Figure S1A). The weak positive correlation suggests that the use of laxatives in constipated patients might have increased moisture contents even in the absence of sufficient fecal mucins.

To further dissect the relationship between mucin and constipation itself, participants were divided into constipated and non-constipated (including laxative-responsive subjects) subgroups. The constipated subgroups exhibited significantly lower fecal mucin content in PD and CIC (Figure 1F). Two-way ANOVA showed that the effect of constipation on fecal mucin contents was significant (*p* = 0.0075), whereas the effect of disease (controls, PD, or CIC) was not (*p* = 0.063), indicating that mucin reduction is more closely associated with constipation rather than disease. As *Akkermansia* is a well-characterized intestinal bacterium that forages mucins,^69^ we analyzed the abundance of *Akkermansia* by 16S rRNA sequencing (Figure 1G) and by qPCR (Figure 1H). Both analyzes showed that *Akkermansia* was increased in CIC and PD compared to controls. *A. muciniphila* is the predominant and well-characterized species in the genus *Akkermansia* in human feces.^70^ Although recent metagenomic analyzes revealed the existence of additional *Akkermansia* species in human feces,^70-72^ we focused on *A. muciniphila* in the following analysis. To further investigate the relationship between fecal parameters and *A. muciniphila* abundance, we divided our samples into two groups of low and high abundances of *A. muciniphila*. As expected, individuals with high *A. muciniphila* had low moisture and mucin contents (Suppl. Figure S1B). Similarly, *A. muciniphila* was higher in constipated subjects than in non-constipated subjects in controls and PD, but not in CIC (Suppl. Figure S1C). As most CIC patients were on laxatives, laxatives might be more effective in patients with high *A. muciniphila* than with low *A. muciniphila*. Alternatively, the causes of CIC were heterogeneous, and a relatively small fraction of CIC patients might be associated with increased *A. muciniphila*.

### Gnotobiotic mice transplanted with *A. muciniphila* show no constipation

**Figure 2.**
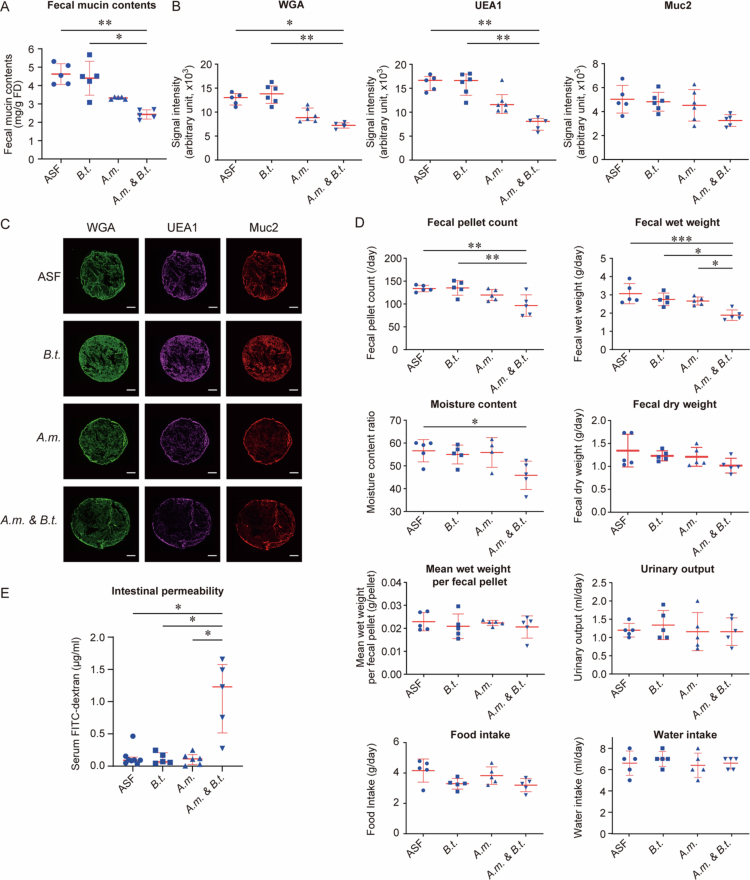
Gnotobiotic mice transplanted with both *A. muciniphila* (*A.m.*) and *B. thetaiotaomicron* (*B.t.*) exhibit constipation and decreased fecal mucus contents. (A) Fecal mucin contents. Quantification (B) and representative images (C) of fecal mucins by fluorescent labeling with WGA, UEA1, and anti-Muc2 antibody. (D) Metabolic features of gnotobiotic mice. Note that fecal pellet counts are comparable to those in previous reports in SPF mice.^81^^,^^82^ (E) Serum FITC-dextran (10 kDa) concentrations at 3 h after its transanal administration to evaluate intestinal permeability. (A, C, D) Mean and SD are indicated. *p < 0.05, **p < 0.01, and ***p < 0.005. One-way ANOVA test followed by Tukey’s multiple comparison test. The normality of the data was assessed using the Shapiro-Wilk test. (E) Median and interquartile range are indicated. *p < 0.05, **p < 0.01, and ***p < 0.005 by Kruskal-Wallis test followed by Dunn’s post hoc test. Altered Schaedler Flora (ASF) was transplanted as a control.

We thus hypothesized that increased *A. muciniphila* may contribute to mucin reduction and consequently to constipation. To test this hypothesis, we generated a gnotobiotic mouse model with *A. muciniphila*. However, the gnotobiotic mice marginally showed constipation phenotypes with slightly reduced fecal pellet counts and slightly reduced fecal moisture contents (Figure 2D). Lack of constipation in our gnotobiotic mice is consistent with a previous study showing a similar lack of constipation in gnotobiotic mice with *A. muciniphila* on a normal diet.^73^ Another study showed that when mice were transplanted with a synthetic set of 14 bacteria including *A. muciniphila*, the mice showed thinning of the mucus layer under a fiber-deprived diet.^74^ As *A. muciniphila* is incapable of degrading sulfated colonic mucins and primarily forages other mucus glycans particularly gastric mucins,^69^ we next searched for colonic mucin-degrading bacteria. A search in the literature identified seven bacteria that were experimentally shown to degrade colonic mucins.^28^^,^^68^^,^^74-80^ Quantification of relative abundances of the seven bacteria by qPCR showed that *A. muciniphila* and *B. thetaiotaomicron* were significantly increased in both PD and CIC (Figure 1H).

### Loperamide-induced constipation fails to increase *A. muciniphila*

To evaluate whether the observed increases in *A. muciniphila* and *B. thetaiotaomicron* in patients with PD and CIC were the cause or the consequence of constipation, we generated a drug-induced constipation model using loperamide in SPF wild-type mice. This drug slows bowel movements by acting on the large intestine.^83^^,^^84^ Loperamide reduced stool output, confirming constipation induction (Suppl. Figure S2A) Among the four glycan-cleaving bacteria detected in our mouse fecal samples, *B. thetaiotaomicron* showed a significant increase in relative abundance after loperamide treatment (Suppl. Figure S2B). In contrast, *A. muciniphila* did not exhibit a statistically significant group-level change, although some mice displayed increased abundance.

To examine whether loperamide directly affected bacterial growth, we conducted *in vitro* growth assays of *A. muciniphila* and *B. thetaiotaomicron* in the presence of 0.0, 0.1, 1.0, and 10 μM loperamide. Loperamide inhibited the growth of *A. muciniphila* but not of *B. thetaiotaomicron* (Suppl. Figure S2C). Thus, the colonic concentration might be too low to suppress the growth of *A. muciniphila*. Alternatively, loperamide suppressed the growth, but unidentified constipation-associated factor(s) might have compensated for the suppressive effect.

We also established another constipation model using a fiber-deprived AIN93M diet (Suppl. Figure S2D). Fiber deprivation had no effect on the abundance of *A. muciniphila* or *B. thetaiotaomicron* (Suppl. Figure S2E).

Lack of increase in *A. muciniphila* in mouse models of loperamide-induced and fiber-deprived constipation points to the notion that its enrichment in PD and CIC patients is unlikely to be a secondary consequence of constipation, but may contribute causally to the mucus degradation and the development of constipation.

We next asked whether laxative use could account for the observed bacterial changes. However, we found that laxative use was dependent on the disease state (controls, CIC, or PD) with the adjusted Cramer's V value of 0.459, and laxatives could not be manipulated as an independent variable to evaluate the effects on bacterial abundances. We additionally asked whether responsiveness to laxative affects bacterial changes. We thus restricted the analysis to CIC patients who were using laxatives and stratified them into non-responders (<3 stools/week) and responders (≥3 stools/week). We found that relative abundances of *A. muciniphila* and *B. thetaiotaomicron* were not statistically different between the two groups (Suppl. Figure S3), indicating that laxative responsiveness is unlikely to account for the increased abundance of these species in CIC patients.

### Gnotobiotic mice transplanted with both *A. muciniphila* and *B. thetaiotaomicron* exhibit constipation

We next generated co-colonized mice by transplanting both *A. muciniphila* and *B. thetaiotaomicron* (*A.m. & B.t.* mice), and compared them with mono-colonized mice transplanted with *A. muciniphila* alone or *B. thetaiotaomicron* alone. The copy numbers of *A. muciniphila* and *B. thetaiotaomicron* per gram of feces were similar between mono- and co-colonized mice, indicating stable colonization in all groups (Suppl. Figure S4). Control gnotobiotic mice were transplanted with ASF, which consisted of eight defined bacteria.^50^ We found that the fecal mucin contents were decreased in *A.m. & B.t.* mice (Figure 2A). Although fecal mucin levels in *A.m.* mice were lower than those in ASF mice, the difference was not statistically significant. Fluorescent staining of fecal mucins by WGA, UEA1, and anti-Muc2 antibody further confirmed decreased fecal mucus levels in *A.m. & B.t.* mice (Figure 2B-C). In contrast, staining in *A.m.* mice did not differ significantly from that in ASF controls, despite an apparent visual decrease.

We next confirmed that the reduced fecal mucins were not due to the reduced mucin production by the colonic epithelial cells in gnotobiotic mice. RNA sequencing analysis of the colonic tissues of mono-colonized (*A.m.* or *B.t.*) and co-colonized (*A.m. & B.t.*) mice showed the expressions of six mucin genes including *Muc2*, four genes for mucin section, and 18 glycosyltranferase genes were not statistically different between the three groups (Suppl. Figure S5). In addition, the numbers and areas of colonic goblet cells were not different between the three gnotobiotic mice (Suppl. Figure S6). Thus, the reduced fecal mucin levels in co-colonized mice were primarily attributable to enhanced mucin-degrading activity by the bacteria.

As expected, fecal analysis with a metabolic cage showed that *A.m. & B.t.* mice had fewer fecal pellets, lower fecal wet weight, and decreased fecal moisture content compared to mono-colonized mice either with *A.m*. or *B.t.*, or ASF mice (Figure 2D). In contrast, food intake, water intake, and urinary volumes remained unchanged in either group (Figure 2D), which made systemic dehydration unlikely as a cause of constipation. In patients with chronic constipation, intestinal permeability was significantly increased, which was evidenced by higher levels of ovalbumin in their serum compared to healthy controls.^85^ We thus examined intestinal permeability by transanal administration of FITC-dextran (10 kDa). After three hours, the plasma level of FITC-dextran was increased only in *A.m & B.t*. mice (Figure 2E), suggesting that the colonic mucus layer was likely to be thinned. Therefore, co-transplantation of *A. muciniphila* and *B. thetaiotaomicron* enhanced mucus foraging, leading to reduced hydration and lubrication of feces as well as increased intestinal permeability, which culminated in constipation.

### Genes in the sulfate assimilation pathway are increased in *A.m.* mice compared to *A.m. & B.t.* mice

**Figure 3.**
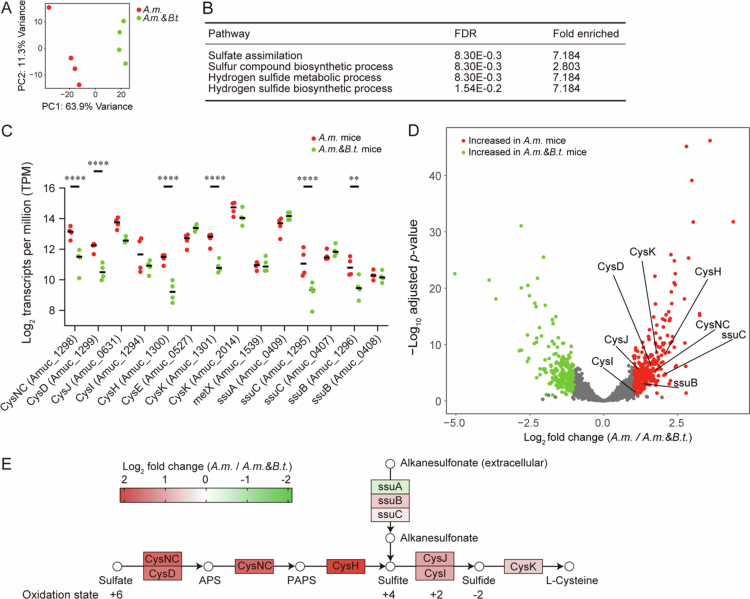
Fecal gene expression profiles of genes encoded in *A. muciniphila* in *A.m.* and *A.m. & B.t.* mice. (A) Principal component analysis (PCA) of quadruplicated fecal RNA-seq of *A.m.* and *A.m.* & *B.t.* mice. The percentage of variance of PC1 and PC2 are indicated. (B) Pathway enrichment analysis with the Gene Ontology Biological Process. Top four pathways are indicated. FDR, false discovery rate. (C) Expressions of 14 genes involved in sulfate metabolisms encoded in *A. muciniphila*. Mean is indicated by a bar. **p < 0.01, ***p < 0.005, and ****p < 0.001 by two-way ANOVA followed by Sidak’s post hoc test. (D) Volcano plot illustrating differential expressions of 2092 genes encoded in *A. muciniphila*. Genes that are significantly enriched in *A.m*. and *A.m.* & *B.t.* mice are indicated in red and green, respectively, where the significance thresholds were set to adjusted *p*-value < 0.1 and |log_2_ fold change| > 1.0. Gene names in the assimilatory sulfate reduction pathway are indicated. (E) KEGG pathways of the assimilatory sulfate reduction and its accessory alkanesulfonate pathway flowing into sulfite are indicated. Enzymes that do not exist in *A. muciniphila* are excluded from the scheme. Differential gene expressions between *A.m.* and *A.m. & B.t.* mice are color-coded. APS, adenosine 5’-phosphosulfate. PAPS, 3’-phosphoadenosine 5’-phosphosulfate.

We next explored why mucus degradation is enhanced in *A.m. & B.t.* mice but not in *A.m.* mice. To this end, feces were subjected to RNA-seq analysis to compare expressions of 2,354 genes encoded in *A. muciniphila* in *A.m.* and *A.m. & B.t.* mice. As expected, RNA-seq reads were not *mapped to B. thetaiotaomicron* genes, because such genes should be absent in *A.m.* mice. Principal component analysis showed that genes expressed in *A.m.* and *A.m. & B.t.* mice made different clusters (Figure 3A). Pathway analysis with the hypergeometric test using the Gene Ontology Biological Process showed that sulfate assimilation, as well as three other biosynthetic processes involving sulfur, were the top four enriched pathways in *A.m.* mice compared to *A.m. & B.t.* mice (Figure 3B). Similarly, inspection of 14 genes associated with sulfur metabolisms in *A. muciniphila* revealed that six genes were significantly increased in *A.m.* mice compared to *A.m. & B.t.* mice (Figure 3C-D). Twelve out of the 14 genes in sulfur metabolisms are in the assimilatory sulfate reduction pathway and its accessory alkanesulfonate pathway in KEGG database (Figure 3E). Color coding of fold-changes in these pathways showed that genes in these pathways except for *ssuA* and *ssuC* were all increased in *A.m.* mice compared to *A.m. & B.t.* mice (Figure 3E). These pathways are part of the bacterial sulfur assimilation machinery, enabling *A. muciniphila* to incorporate inorganic or alternative sulfur sources into biomolecules. However, it is important to note that these pathways are functionally distinct from sulfatase activity, which is required to hydrolytically cleave sulfate moieties from complex sulfated glycans, such as colonic mucins. One possible explanation for the increased sulfate assimilation pathway in *A.m.* mice is that sulfate assimilation genes were upregulated to compensate for a lack of sulfate. Alternatively, lack of sulfate in the absence of *B. thetaiotaomicron* might have excessively stimulated host intestinal epithelial cells to provide a sufficient amount of sulfate for *A. muciniphila*.

### Activation of sulfatases in *B. thetaiotaomicron* is a key to cause constipation in *A.m. & B.t.* mice

We next asked whether the sulfatases are indeed keys to degrade fecal mucins in *A.m. & B.t.* mice. The anaerobic sulfatase-maturating enzyme (anSME) in *B. thetaiotaomicron* is critical in the post-translational activation of sulfatases.^66^^,^^86^ anSME is a radical S-adenosylmethionine (SAM) enzyme that catalyzes the post-translational modification of a cysteine or serine residue into Cα-formylglycine. This modification is essential for activating sulfatases, which enables bacteria such as *B. thetaiotaomicron* to degrade sulfated glycans, including mucin O-glycans, and utilize them as nutrient sources.^65^ A previous report showed that knockout of *anSME* in *B. thetaiotaomicron* led to a loss of sulfatase activity and impairs the ability to utilize sulfated polysaccharides as a carbon source.^31^ We thus knocked out the gene encoding anSME in *B. thetaiotaomicron* by homologous recombination (Suppl. Figure S7). We confirmed lack of sulfatases in the Δ*anSME* strain by growing them under sulfated glycans (chondroitin sulfate or heparin) as the only carbon source. As expected, the Δ*anSME* strain could grow in minimum medica with 0.5% glucose, but not with 0.5% chondroitin sulfate or 0.5% heparin (Suppl. Figure S8). These results indicate that *anSME* is essential for the degradation and utilization of sulfated glycosaminoglycans.

**Figure 4.**
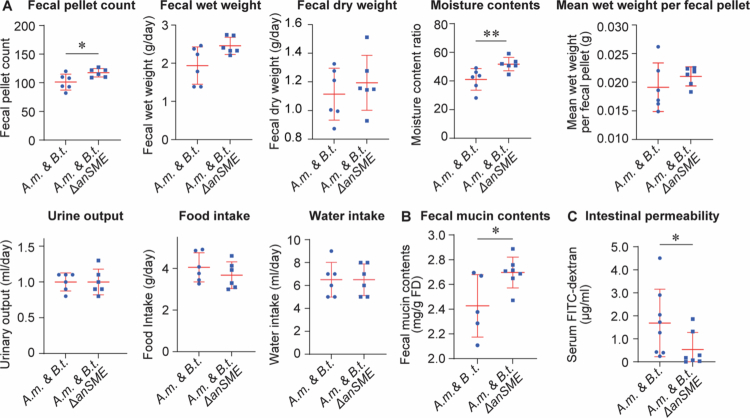
*A.m. & B.t.ΔanSME* mice lacking the anaerobic sulfatase-maturating enzyme (anSME) in *B. thetaiotaomicron* ameliorates constipation in *A.m. & B.t.* mice. Metabolic features (A) and fecal mucin contents (B) of *A.m.* & *B.t.* and *A.m.* & *B.t.*Δ*anSME* mice. (C) Serum FITC-Dextran (10 kDa) concentrations at 3 h after its transanal administration to evaluate intestinal permeability. Mean and SD are indicated. *p < 0.05 and **p < 0.01 by Mann-Whitney test.

We then generated gnotobiotic mice transplanted with wild-type strain of *A. muciniphila* and the *anSME*-deficient strain of *B. thetaiotaomicron* (*A.m. & B.t.*Δ*anSME* mice). Quantification of bacterial genomic copy numbers in fecal samples showed that the Δ*anSME* strain was able to colonize to a similar level to wild-type strain, indicating that the deletion of *anSME* did not affect the colonization of *B. thetaiotaomicron* (Suppl. Figure S9). We found no statistically significant difference in fecal colonization levels between the wild-type and Δ*anSME* strains, indicating that the Δ*anSME* mutant retains colonization ability comparable to the wild-type strain. Evaluation of constipation by a metabolic cage and a freeze-dryer revealed that both fecal pellet counts and moisture contents were increased in *A.m. & B.t.*Δ*anSME* mice compared to *A.m. & B.t.* mice (Figure 4A). Similarly, fecal mucin contents were increased (Figure 4B) and intestinal permeability was decreased (Figure 4C) in *A.m. & B.t.*Δ*anSME* mice. These results indicate that sulfatases activated by anSME are essential for efficient mucin degradation in *A.m. & B.t.* mice. Thus, lack of functional sulfatases in *B. thetaiotaomicron* led to impaired mucus degradation, preserved fecal hydration and intestinal permeability, which protected the development of constipation in *A.m. & B.t.*Δ*anSME* mice.

## Discussion

Metagenomic analysis of PD, CIC, and controls (Figure 1G), as well as qPCR of seven mucin-degrading bacteria (Figure 1H) pointed to *A. muciniphila* and *B. thetaiotaomicron* as candidates that are associated with constipation. Although additional species were recently identified in genus *Akkermansia*, ^71^^,^^72^ *A. muciniphila* is a dominant species in human intestine.^70^ The increase of *A. muciniphila* was in accordance with previously reported 16S rRNA sequencing data in patients with slow gut transit time.^15-17^^,^^87^ Gnotobiotic mice carrying either *A. muciniphila* or *B. thetaiotaomicron* alone failed to develop constipation (Figure 2). Lack of constipation in our *A.m.* mice was consistent with a previous report showing that gnotobiotic mice with *A. muciniphila* showed no effect on intestinal mucins.^73^ *A. muciniphila* is able to forage mucins with terminal sialyl and fucosyl residues, but not with terminal sulfates.^88^ As colonic mucins are enriched in terminal sulfates, *A. muciniphila* was likely to have failed to digest colonic mucins. In contrast, gnotobiotic mice co-transplanted with both bacteria (*A.m. & B.t.* mice) developed constipation. As *B. thetaiotaomicron* provides sulfatases to remove terminal sulfates from colonic mucins, *A. muciniphila* was able to completely degrade colonic mucins and induced constipation in *A.m. & B.t.* mice (Figure 4). A previous study showed that gnotobiotic mice transplanted with a synthetic set of 14 bacteria showed thinning of the mucus layer under a fiber-deprived diet.^74^ As both *A. muciniphila* and *B. thetaiotaomicron* were included in the 14 bacteria, the two bacteria were likely to have exerted substantial effects on the thinning of the mucus layer.

Fecal RNA-seq analysis of genes encoded in *A. muciniphila* in *A.m. & B.t.* and *A.m.* mice revealed that sulfate assimilation pathway was upregulated in *A.m.* mice compared to *A.m. & B.t.* mice (Figure 3). Although the upregulated sulfate assimilation pathway in *A.m.* mice was totally unexpected, we next examined whether sulfatases in *B. thetaiotaomicron* were indeed critical in the development of constipation in *A.m. & B.t.* mice. We thus deleted *anSME*, an essential gene for maturation of sulfatases, in *B. thetaiotaomicron* by homologous recombination (Suppl. Figure S7). As expected, *A.m. & B.t.*Δ*anSME* mice developed milder constipation, increased fecal mucin contents, and decreased intestinal permeability compared to those in *A.m. & B.t.* mice (Figure 4). Sulfatases in *B. thetaiotaomicron* potentially exert their effects on *A. muciniphila*. First, as has been repeatedly stated, sulfatases remove the terminal sulfate of colonic mucins,^31^ so that *A. muciniphila* is able to forage colonic mucins. Second, sulfatases provide sulfate for *A. muciniphila*, which is reduced to sulfide to synthesize L-cysteine and its downstream sulfide-containing molecules (Figure 4E). *Desulfovibrio* spp. are sulfate-reducing bacteria (SRB), and require sulfate for their growth, as they use sulfate as a terminal electron acceptor in their energy metabolism through dissimilatory sulfate reduction.^89^ Similar to *A. muciniphila*, *Desulfovibrio* spp. are dependent on sulfate generated by other bacteria. It is interesting to note that both *A. muciniphila*^11^^,^^90-95^ and *Desulfovibrio* spp.^96-99^ are increased in PD patients. Sufficient supply of sulfate by *B. thetaiotaomicron*, which was also increased in PD patients (Figure 1H), may partly account for the increases of *A. muciniphila* and *Desulfovibrio* spp.

*A. muciniphila* is a mucin-specialist bacterium capable of using host-derived mucins as its sole carbon.^74^^,^^80^^,^^100^ In contrast, sulfatases are not unique in *B. thetaiotaomicron.* Other bacteria including *B. caccae* and *B. uniformis* in *Bacteroides* spp. are capable of generating sulfatases and of substituting for *B. thetaiotaomicron* in providing sulfatases.^31^ The redundancy of sulfatase-providing bacteria also supports the notion that *A. muciniphila* is a key bacterium to drive mucus depletion although collaborating bacteria are required for its full functionality.

Our study has the following limitations. First, although CIC frequently precedes the development of PD, we assumed that fractions of patients with CIC and PD share a similar bacterial origin of constipation. We indeed found that *A. muciniphila* and *B. thetaiotaomicron* were increased in both CIC and PD. However, additional unidentified bacteria may also exert similar constipation-inducing effects. Second, the sample sizes were not matched between controls, PD, and CIC. A large sample size of PD patients could readily yield statistical significance, whereas a small sample size of CIC patients could not. We suppose that this is a commonly recognized limitation in exploratory clinical studies. Third, we could not perform immunohistochemical analyzes of fecal mucins and the Muc2 protein in human samples, because frozen stool samples did not keep microscopic integrity. Instead, we quantified mucin-derived O-glycans in human samples, and found that they were indeed decreased in in patients with constipation, as well as in patients with higher abundance of *A. muciniphila* (Figure 1). Finally, while we showed *A. muciniphila* and *B. thetaiotaomicron* cooperatively induced desulfation and degradation of colonic mucins and induced constipation, sulfated glycans and mucin-degrading activities were not quantified by us or others in fecal samples in constipated patients because of technical difficulty.^101^^,^^102^ However, shotgun-metagenomic analyzes of human fecal samples were in accordance with our murine observations. First, a microbial CAZyme repertoire was biased toward the mucin degradation rather than the dietary fiber degradation and mucin-degrading taxa were increased in patients with PD accompanied by REM sleep behavior disorder.^103^ Second, genes for sulfuric ester hydrolase (KEGG Orthology: KO1138, EC 3.1.6) that degrades sulfated mucin glycans were increased in a large cohort of PD patients.^104^

In conclusion, we showed that *A. muciniphila* is a key bacterium in the development of constipation, and another sulfatase-providing bacterium plays an accessory but indispensable role in its development. Both bacteria coordinately deplete colonic mucins, which subsequently reduces water preservation and mucus lubrication. Our study suggests the presence of bacterial constipation in humans. Fecal abundance of *A. muciniphila* may serve as a biomarker for identifying such patients. In addition, phage-mediated bacterial suppression or small molecules to block bacterial sulfatases may preserve colonic mucus integrity, improve stool hydration, and alleviate constipation in these patients.

## Supplementary Material

Supplementary Material Legends_for_Supplementary_Figures_clean

## Data Availability

Raw fecal RNA sequences were deposited to DDBJ with BioProject ID PRJDB19181. Raw 16S rRNA sequences of CIC were deposited to DDBJ with BioProject ID PRJDB20342. Raw 16S rRNA-sequences of controls and PD were previously deposited to DDBJ with BioProject ID PRJDB8639. Raw colonic RNA sequences were deposited to DDBJ with BioProject PRJDB35762. Any additional information required to reanalyze the data reported in this paper is available from the lead contact upon request.

## Material availability

The *anSME*-deficient strain of *B. thetaiotaomicron* is available upon request.
