# Supplementary material for: Bacterial constipation: Mucin-degrading intestinal commensal bacteria cause constipation
Source: Gut Microbes. 2026 Feb 18;18(1):2596809. doi: 10.1080/19490976.2025.2596809 (PMC12928629; doi:10.1080/19490976.2025.2596809)
Supplement: Supplementary Material — Legends_for_Supplementary_Figures_clean [file KGMI_A_2596809_SM2347.docx]

**
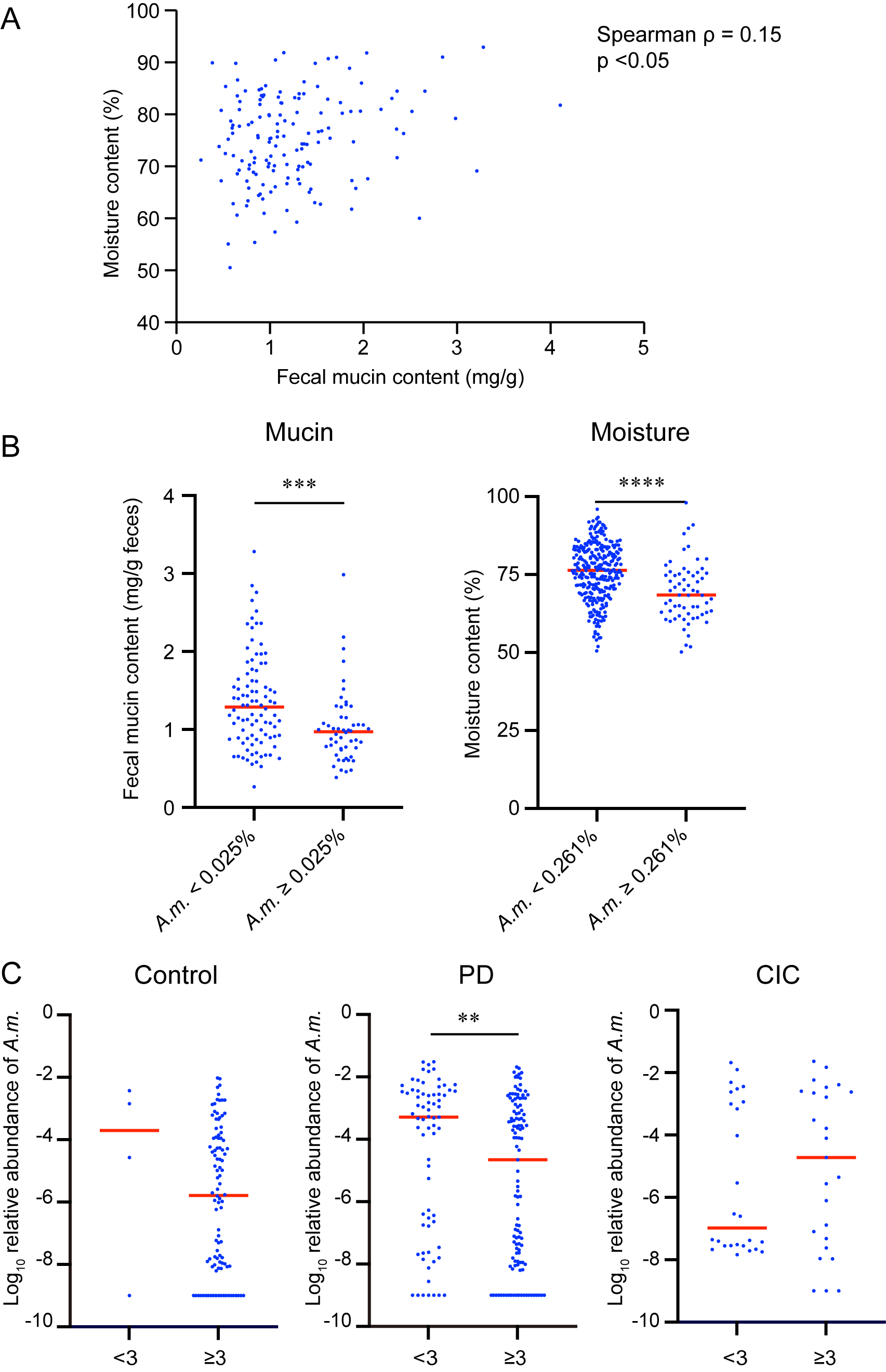
**

**Supplementary Figure S1. Correlation analyses between fecal mucin content, fecal moisture content, and relative abundance of *A. muciniphila (A.m.)* in controls, PD, and CIC**

**A** Scatter plot of fecal mucin content (mg/g) and fecal moisture content (%) of a combined cohort of controls, PD, and CIC. The ρ value by Spearman’s correlation analysis is indicated (*n* = 151).

**B** Fecal mucin contents (*n* = 144) and moisture contents (*n* = 350) in two categories of low and high abundances of *A. m.* in a combined cohort of controls, PD, and CIC*.* The thresholds of *A.m.* were determined by the partitioning functionality of JMP pro to make decision trees.

**C** Relative abundances of *A.m.* in constipated and non-constipated subgroups in controls (*n* = 104), PD (*n* = 193), and CIC (*n* = 51).

**B, C** Medians are indicated in red. ^**^*P* < 0.01, ^***^*P* < 0.005, and ^****^*P* < 0.001 by Mann-Whitney test.

**
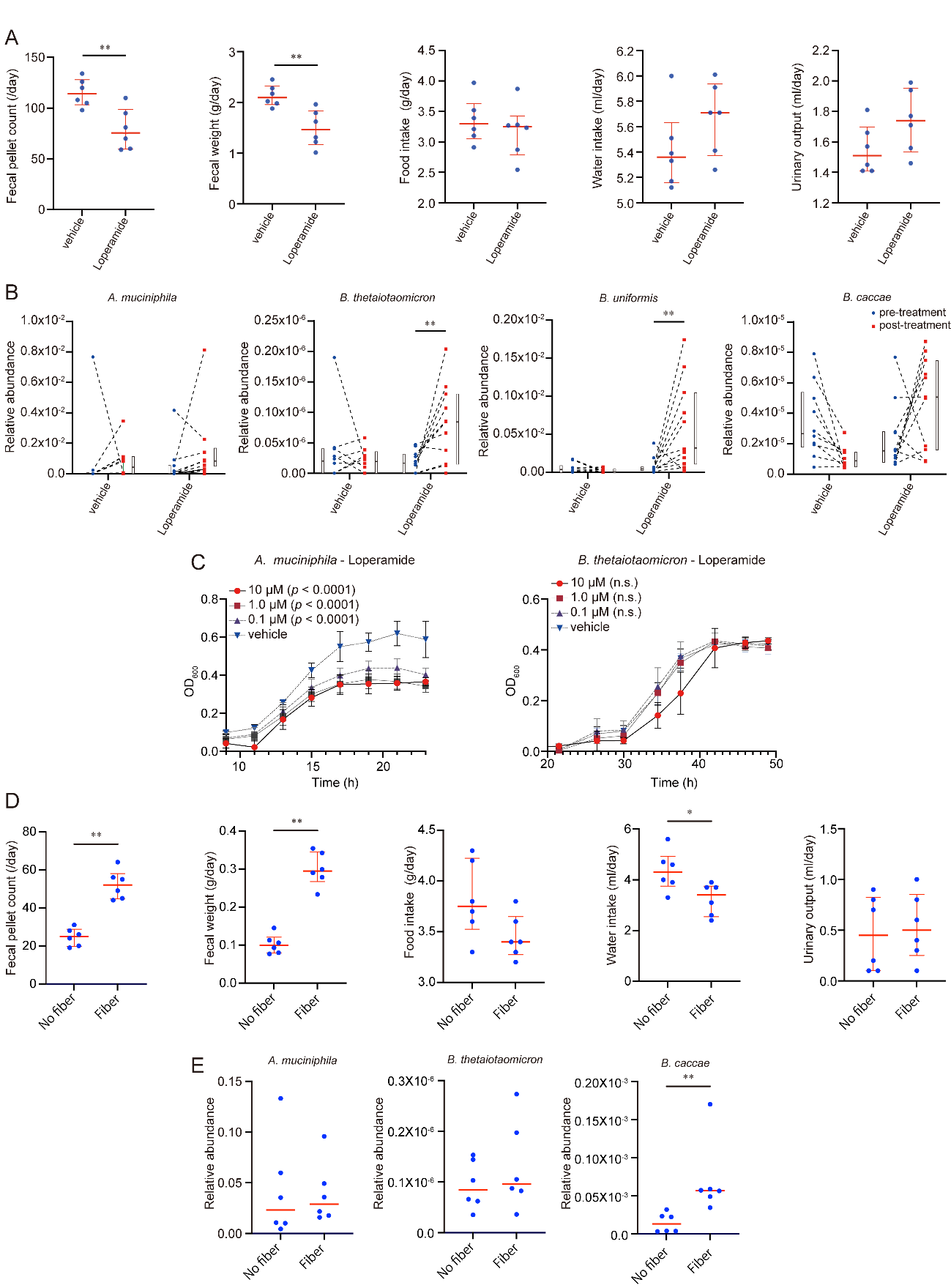
**

**Supplementary Figure S2. Loperamide-induced and fiber-deprivation-induced constipation models in SPF wild-type mice and their effects on *A. muciniphila in vitro* or *in vivo***

**A** Metabolic feature of the mouse model of loperamide-induced constipation (n=6). Median and interquartile range are indicated. ***p* < 0.01 by Mann-Whitney test.

**B** Relative abundances of four indicated bacteria before and after six weeks of loperamide treatment quantified by qPCR. Out of the seven experimentally proven glycan-cleaving intestinal bacteria, the four indicated bacteria were present in our mouse fecal samples. Medians and interquartile range are indicated. ***p* < 0.01 by restricted maximum likelihood (REML) accounting for paired measurements.

**C** *In vitro* growth curves of *A. muciniphila* and *B. thetaiotaomicron* in the presence of loperamide. Growth was monitored at OD₆₀₀ over time in cultures containing vehicle control or 0.1–10 μM loperamide. Mean and SD (*n* = 6) are indicated. Statistical significance compared to vehicle by two-way repeated measures ANOVA followed by Dunnett’s post hoc test is indicated in the legend.

**D** Metabolic measures in SPF C57BL/6N male mice fed with fiber-deprived (*n* = 6 mice) and fiber-supplemented (*n* = 6 mice) diets for 1 week.

**E** Relative abundances of the three indicated bacteria after one week of dietary intervention quantified by qPCR. Median is indicated. ***p* < 0.01 by Mann-Whitney test.


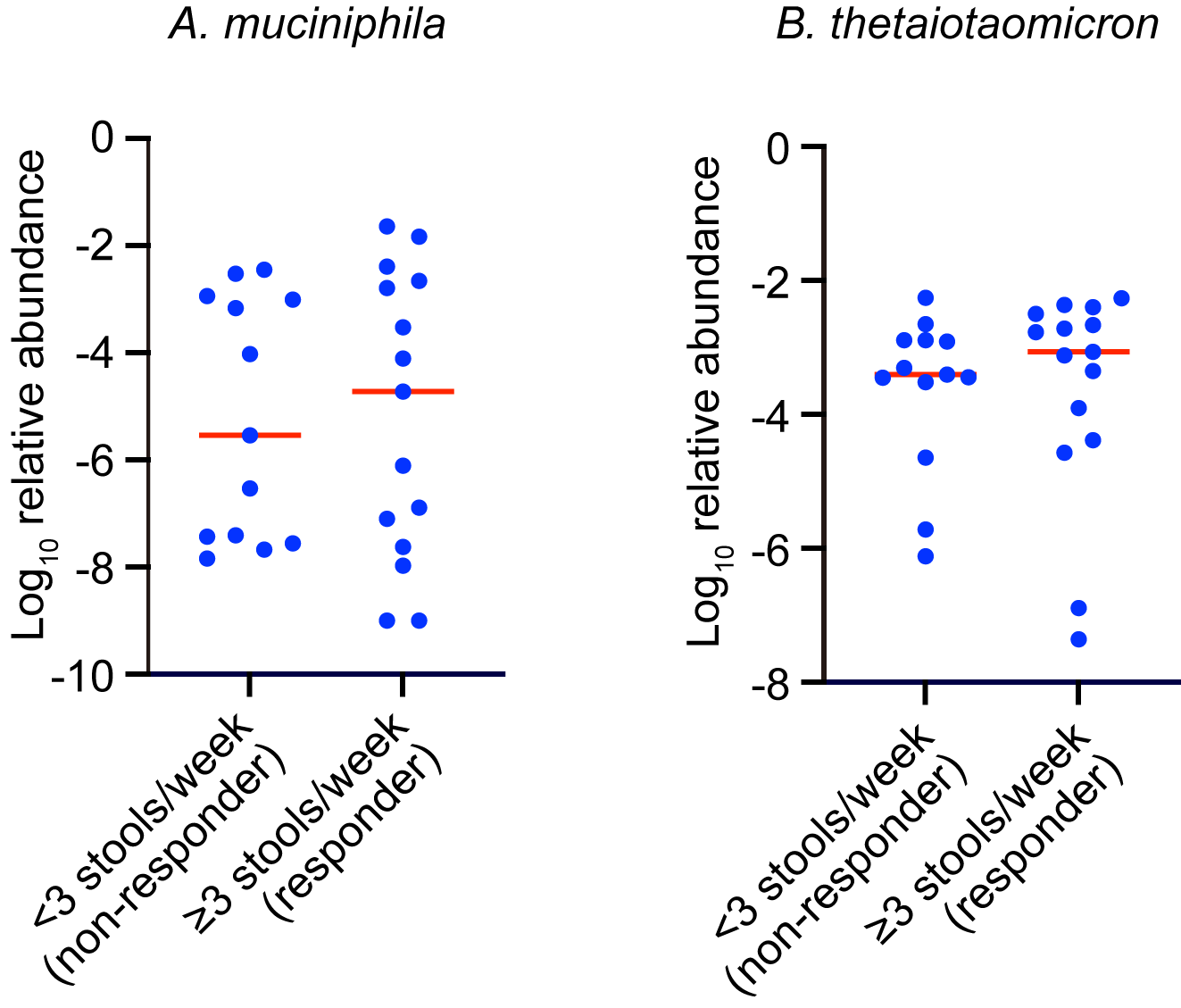


**Supplementary Figure S3. Effect of laxative response status on *A. muciniphila* and *B. thetaiotaomicron* abundance in CIC patients using laxatives**

Relative abundances of *A. muciniphila* and *B. thetaiotaomicron* in CIC patients using laxatives, stratified by stool frequency into non-responders (<3 stools/week, n = X) and responders (≥ three stools/week, n = Y). Medians are indicated. Statistical comparisons were performed using the Mann–Whitney test; no statistically significant differences were detected for either species.

**
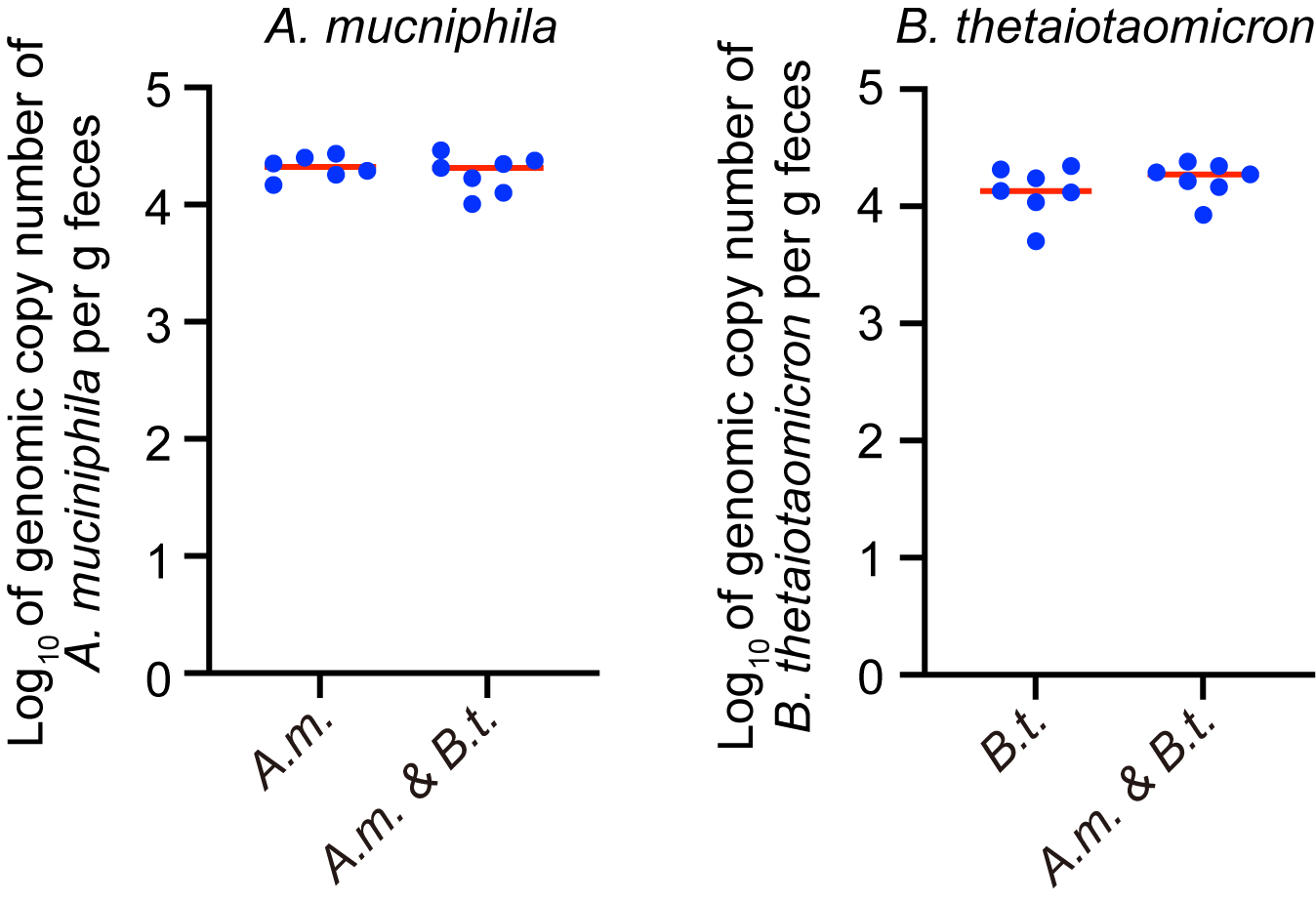
**

**Supplementary Figure S4. qPCR analysis to assess bacterial colonization in gnotobiotic mice**

Genomic copy number of *A. muciniphila* and *B. thetaiotaomicron* per gram of feces in recipient mice following fecal microbiota transplantation. Medians are indicated. No statistical significance between mono- and co-colonized mice by Mann–Whitney test.


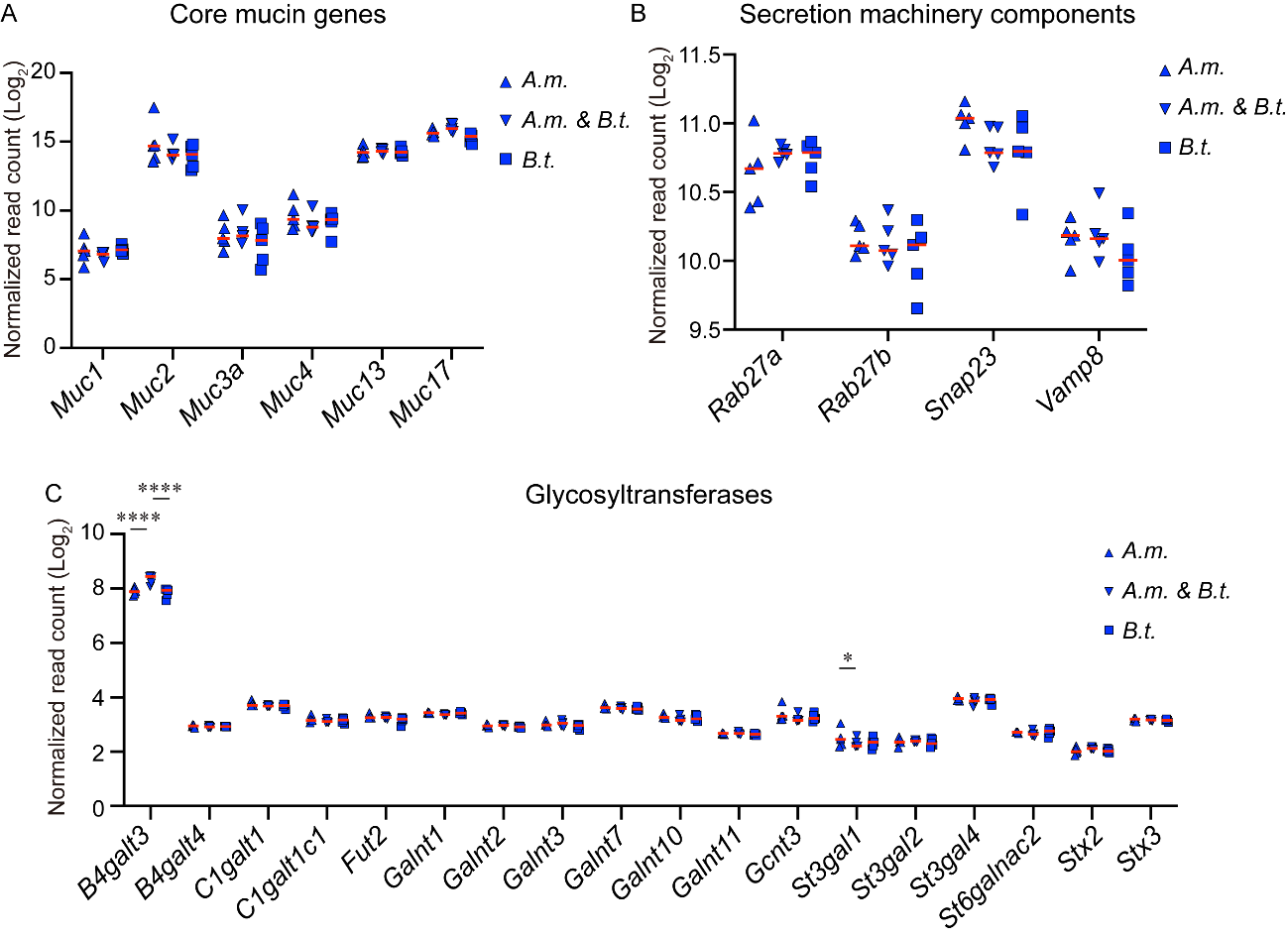


**Supplementary Figure S5. Expression profiles of colonic tissues of genes for core mucins (A), secretion machinery components (B), and glycosyltransferases (C) in *A.m., B.t.,* and *A.m. & B.t.* mice**

Medians are indicated (*n* = 5). **P* < 0.05 and *****P* < 0.001 by Kruskal–Wallis test followed by Dunnett’s posthoc test.

**
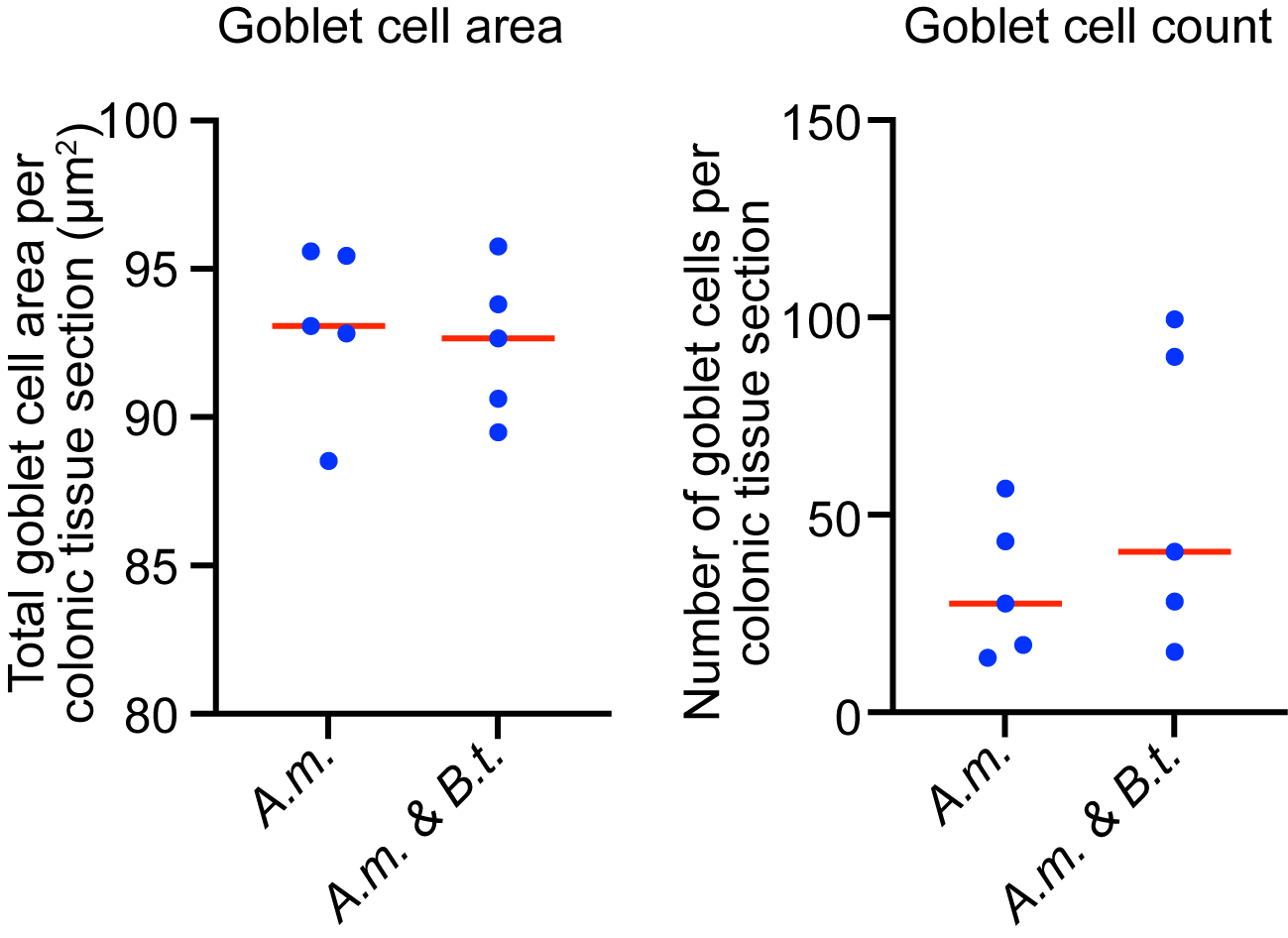
**

**Supplementary Figure S6. The area (A) and the number (B) of goblet cells per colonic tissue section in *A.m.* and *A.m. & B.t.* mice**

**A** Median goblet cell areas per colonic section are plotted (*n* = 5 mice)

**B** Number of goblet cells per colonic section are plotted (*n* = 5 mice)

Images were analyzed using Fiji. No statistical difference by Mann-Whitney test.


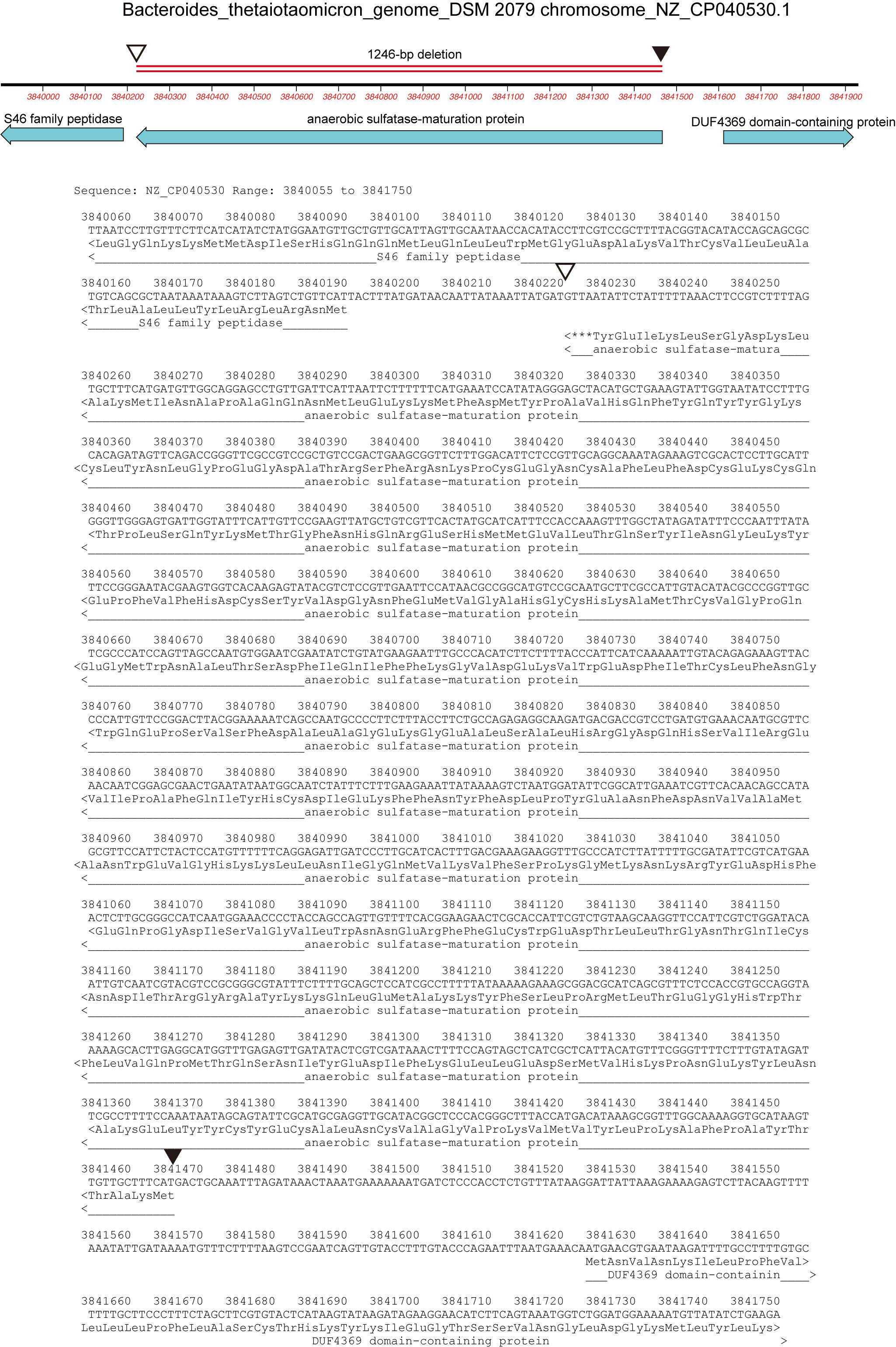


**Supplementary Figure S7. Position of the deletion of the anaerobic sulfatase-maturating enzyme gene (*anSME*) in *B. thetaiotaomicron* (strain DSM 2079)**

A deletion (1246 bp) of *anSME* from positions 3,840,221 to 3,841,466 (NZ_CP040530.1) was introduced by homologous recombination. The start and the end of the deletion are indicated by open and closed arrowheads, respectively.


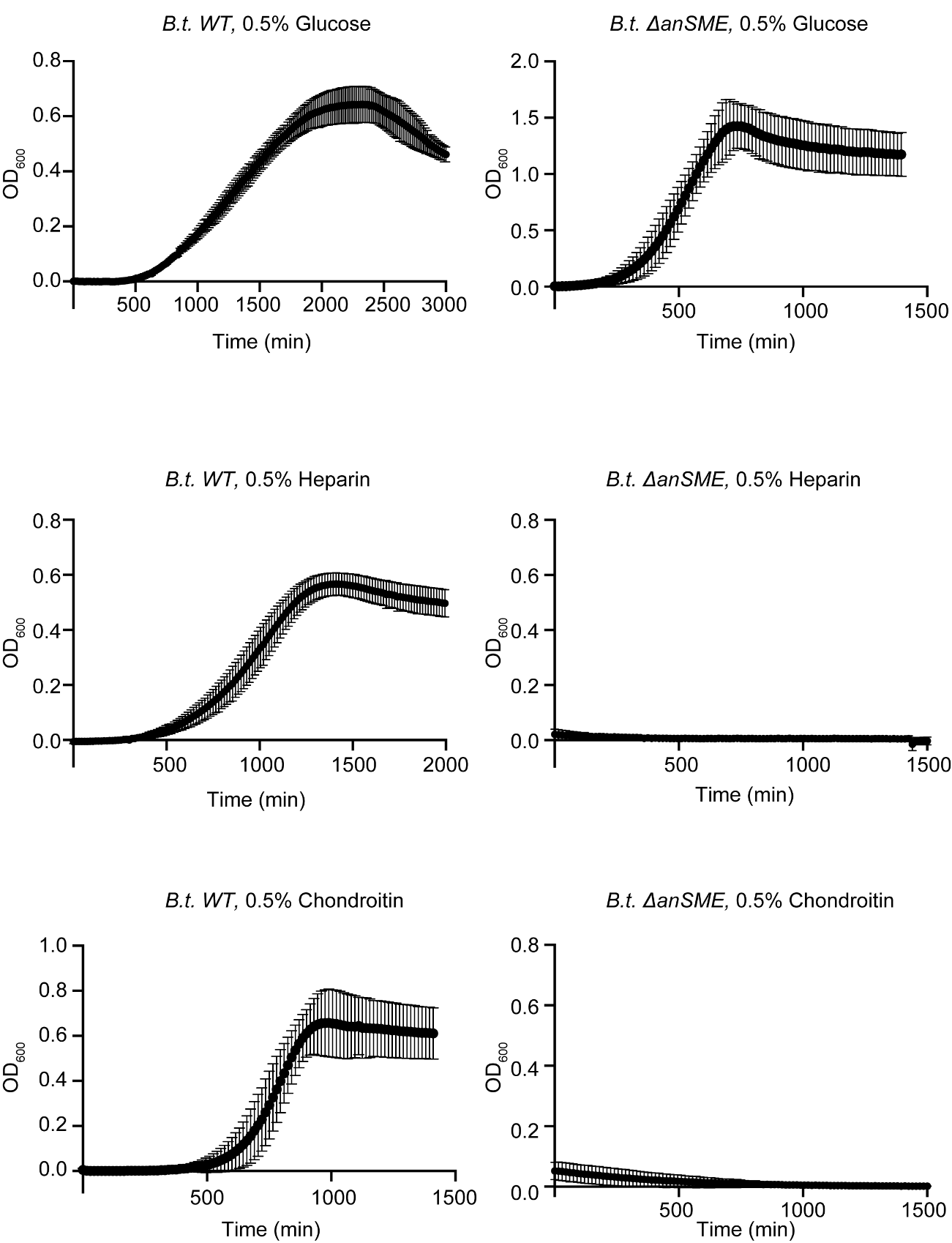


**Supplementary Figure S8. Deletion of *anSME* abolishes growth on sulfated glycans**

Growth curves of wild-type *B. thetaiotaomicron* (*B.t.* WT) and *ΔanSME* mutant (*B.t. ΔanSME*) in three different media (0.5% glucose, 0.5% heparin, and 0.5% chondroitin). Data represent mean ± SD (*n* = 3 culture tubes).

**
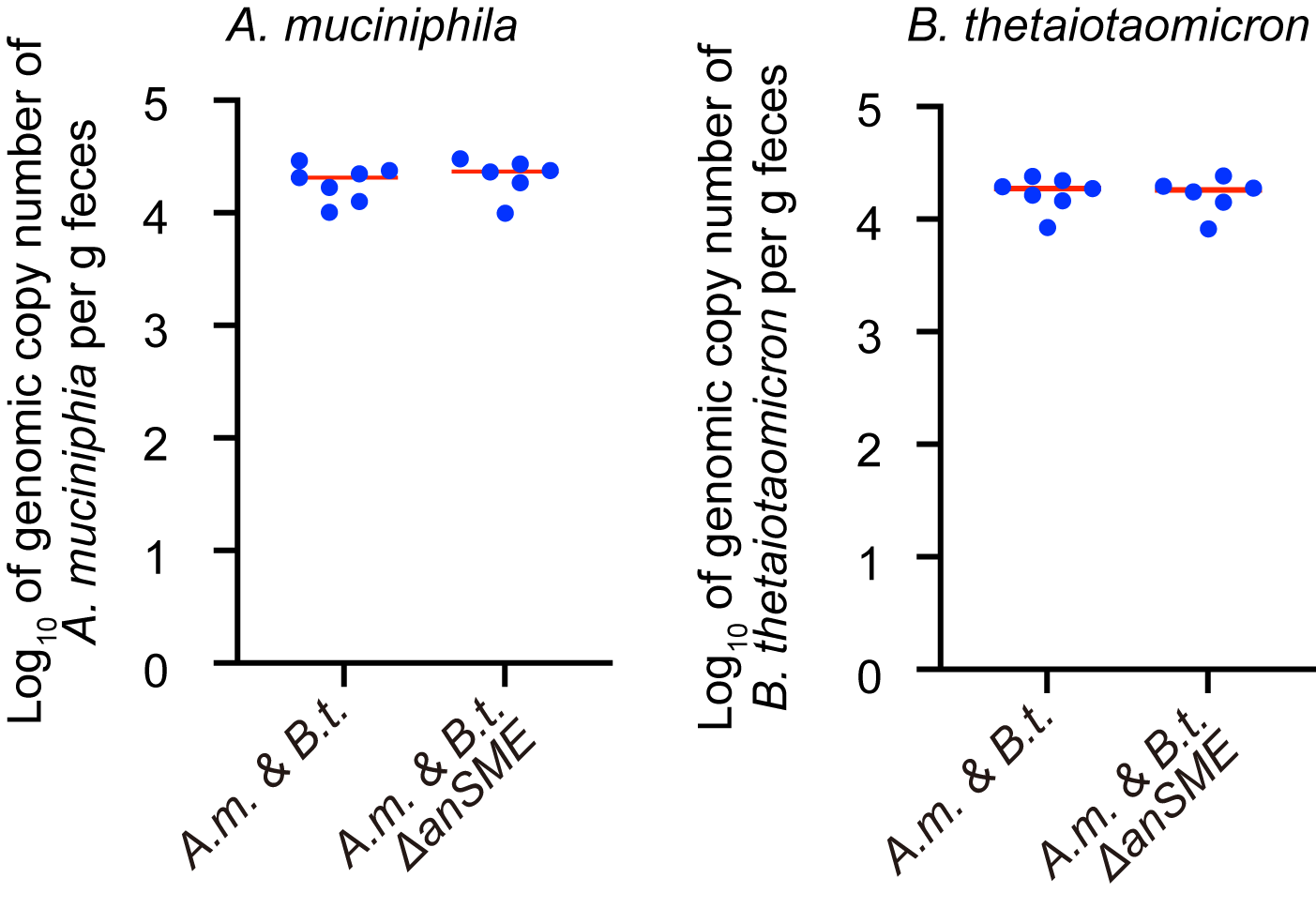
**

**Supplementary Figure S9. Deletion of *anSME* does not affect colonic colonization in gnotobiotic mice**

Copy numbers of *A. muciniphila* and *B. thetaiotaomicron* per gram of feces in gnotobiotic mice co-colonized with *A.m & B.t.* and *A.m. & B.t.ΔanSME* are plotted. Medians are indicated. No statistical difference by Mann–Whitney test.

**Supplementary Table S1. Primers and probes for qPCR**

| Bacterial species | Forward primer (5'-3') | Reference |
| --- | --- | --- |
|  | Reverse primer (5'-3') |  |
|  | Probe (5'-3') |  |
| All bacteria | TCCTACGGGAGGCAGCAGT | ^1^ |
| (universal 16S rDNA) | GGACTACCAGGGTATCTAATCCTGTT |  |
|  | CGTATTACCGCGGCTGCTGGCAC |  |
| *A. muciniphila* | CGGTGGAGTATGTGGCTTAAT | ^2^ |
|  | CCATGCAGCACCTGTGTAA |  |
|  | CGCCTCCGAAGAGTCGCATG |  |
| *B. caccae* | AAACCCATACGCCGCAAG | ^3^ |
|  | GACACCTCACGGCACGAG |  |
|  | TGTGAAGGTGCTGCATGGTTGTCGT |  |
| *B. fragilis* | TCRGGAAGAAAGCTTGCT | ^3^ |
|  | CATCCTTTACCGGAATCCT |  |
|  | ACACGTATCCAACCTGCCCTTTACTCG |  |
| *B. thetaiotaomicron* | GCAAACTGGAGATGGCGA | ^3^ |
|  | AAGGTTTGGTGAGCCGTTA |  |
|  | TCGATGGGGATGCGTTCCATTAGG |  |
| *P. vulgatus* | CGGGCTTAAATTGCAGATGA | ^3^ |
|  | CATGCAGCACCTTCACAGAT |  |
|  | TGAAAGCCGTAAGCCGCAAGG |  |
| *B. longum* | TGGAAGACGTCGTTGGCTTT | ^4^ |
|  | ATCGCGCCAGGCAAAA |  |
|  | CGCACCCACCGCA |  |
| *R. torques* | GCTTAGATTCTTCGGATGAAGAGGA | ^5^ |
|  | AGTTTTTACCCCCGCACCA |  |
|  | CTGTATGAGGCAGGTTACCCACGC |  |

**Supplementary Table S2. PCR primers for generation of an *anSME*-deletion mutant of *B. thetaiotaomicron***

| Primer name | Forward primer (5'-3') |
| --- | --- |
|  | Reverse primer (5'-3') |
| anSME-upstream-F | ﻿AATACGGATTTTATCGCTGGCGTTCATCACTTCTTT |
| anSME-upstream-R | ﻿CATCATAATTTATAATTGTTATCATAAAGTAATGAA |
| anSME-downstream-F | ﻿GACTGCAAATTTAGATAAACTAAATGAAAAAAATGA |
| anSME-downstream-R | ﻿CAGGAAACTGCCAAAAAAGAAAGAATAAAGAAAAAA |
| In-Fusion-upstream-F | ﻿ATTATGAGTGGATCCAATACGGATTTTATCGCTGG |
| In-Fusion-upstream-R | ﻿TGCAGTCCATCATAATTTATAATTGTTATCAT |
| In-Fusion-downstream-F | ﻿TTATGATGGACTGCAAATTTAGATAAACTA |
| In-Fusion-downstream-R | ﻿CAGCCCGGGGGATCCCAGGAAACTGCCAAAAAAGA |

**References**

1. Nadkarni MA, Martin FE, Jacques NA, Hunter N. Determination of bacterial load by real-time PCR using a broad-range (universal) probe and primers set. Microbiology 2002; 148:257–66.

2. Liu S, Rezende RM, Moreira TG, Tankou SK, Cox LM, Wu M, Song A, Dhang FH, Wei Z, Costamagna G, et al. Oral administration of miR-30d from feces of MS patients suppresses MS-like symptoms in mice by expanding Akkermansia muciniphila. Cell Host Microbe 2019; 26:779-794.e8.

3. Tong J, Liu C, Summanen P, Xu H, Finegold SM. Application of quantitative real-time PCR for rapid identification of Bacteroides fragilis group and related organisms in human wound samples. Anaerobe 2011; 17:64–8.

4. Haarman M, Knol J. Quantitative real-time PCR assays to identify and quantify fecal Bifidobacterium species in infants receiving a prebiotic infant formula. Appl Environ Microbiol 2005; 71:2318–24.

5. Png CW, Lindén SK, Gilshenan KS, Zoetendal EG, McSweeney CS, Sly LI, McGuckin MA, Florin THJ. Mucolytic bacteria with increased prevalence in IBD mucosa augment in vitro utilization of mucin by other bacteria. Am J Gastroenterol 2010; 105:2420–8.
